# Cognitive activity significantly affects the dynamic cerebral autoregulation, but not the dynamic vasoreactivity, in healthy adults

**DOI:** 10.3389/fphys.2024.1350832

**Published:** 2024-09-09

**Authors:** Jasmin M. Rizko, Lucy C. Beishon, Ronney B. Panerai, Vasilis Z. Marmarelis

**Affiliations:** ^1^ A. E. Mann Department of Biomedical Engineering, University of Southern California, Los Angeles, CA, United States; ^2^ Cerebral Haemodynamics in Ageing and Stroke Medicine Research Group, Department of Cardiovascular Sciences, University of Leicester, Leicester, United Kingdom; ^3^ Leicester Biomedical Research Centre, National Institute for Health Research, Leicester, United Kingdom

**Keywords:** cerebral blood velocity, dynamic cerebral autoregulation, dynamic vasomotor reactivity, neurovascular coupling, transcranial doppler ultrasonography

## Abstract

**Introduction:**

Neurovascular coupling (NVC) is an important mechanism for the regulation of cerebral perfusion during intensive cognitive activity. Thus, it should be examined in terms of its effects on the regulation dynamics of cerebral perfusion and its possible alterations during cognitive impairment. The dynamic dependence of continuous changes in cerebral blood velocity (CBv), which can be measured noninvasively using transcranial Doppler upon fluctuations in arterial blood pressure (ABP) and CO_2_ tension, using end-tidal CO_2_ (EtCO_2_) as a proxy, can be quantified via data-based dynamic modeling to yield insights into two key regulatory mechanisms: the dynamic cerebral autoregulation (dCA) and dynamic vasomotor reactivity (DVR), respectively.

**Methods:**

Using the Laguerre Expansion Technique (LET), this study extracted such models from data in supine resting vs cognitively active conditions (during attention, fluency, and memory tasks from the Addenbrooke’s Cognitive Examination III, ACE-III) to elucidate possible changes in dCA and DVR due to cognitive stimulation of NVC. Healthy volunteers (n = 39) were recruited at the University of Leicester and continuous measurements of CBv, ABP, and EtCO_2_ were recorded.

**Results:**

Modeling analysis of the dynamic ABP-to-CBv and CO_2_-to-CBv relationships showed significant changes in dCA, but not DVR, under cognitively active conditions compared to resting state.

**Discussion:**

Interpretation of these changes through Principal Dynamic Mode (PDM) analysis is discussed in terms of possible associations between stronger NVC stimulation during cognitive tasks and enhanced sympathetic activation.

## 1 Introduction

Under normal conditions, cerebral blood flow and neuronal activity are closely linked through a process known as neurovascular coupling (NVC) (Phillips et al., 2016; Iadecola, 2017; Hosford and Gourine, 2019; Hosford et al., 2022). NVC is mediated through the neurovascular unit which comprises neurons, endothelial cells, astrocytes, perivascular smooth muscle, and pericytes. A rise in blood CO_2_ tension is also known to cause vasodilation and increased cerebral blood flow through the physiological regulatory mechanism of dynamic vasomotor reactivity (DVR) (Ringelstein et al., 1992; Maeda et al., 1993; Carlson et al., 2008; Ainslie and Duffin, 2009; Battisti-Charbonney et al., 2011). Together, NVC and DVR adjust cerebral blood flow to match localized neuronal metabolic demands. This serves to ensure the delivery of key substrates for neuronal metabolism (e.g., oxygen, glucose), but also to remove the metabolic waste products that are neurotoxic if left to accumulate (Phillips et al., 2016; Hosford and Gourine, 2019). NVC has been shown to be impaired in a range of conditions, for example, stroke and dementia (Beishon et al., 2017; Beishon et al., 2018a; Beishon L. et al., 2018; Salinet et al., 2019; Beishon et al., 2021b; Beishon L. C. et al., 2021). Thus, NVC dysfunction is a potential therapeutic target and has become the focus of increasing research interest (Phillips et al., 2016). Likewise, DVR has been shown to be impaired in diabetes (Novak et al., 2011), Alzheimer’s disease (Marmarelis et al., 2013), mild cognitive impairment (Van Dijk et al., 2007; Marmarelis et al., 2017; Marmarelis et al., 2020), hypertension (Marmarelis et al., 2019), ischemia (Govindan et al., 2019), and Parkinson’s disease (Barnes et al., 2022).

In addition to NVC and DVR, there exists another regulatory mechanism that seeks to buffer the cerebral flow response to abrupt changes of ABP, known as dynamic cerebral autoregulation (dCA) (Aaslid, 2006; Carlson et al., 2008; Panerai, 2008; Panerai, 2009; Liu et al., 2015; Claassen et al., 2016; Claassen et al., 2021; Placek et al., 2017; Silverman and Petersen, 2023; Panerai et al., 2023). The dCA mechanism protects the cerebral vasculature from rapid surges of ABP and prevents hypoperfusion during sudden ABP drops (Aaslid et al., 1989). We have previously investigated the interaction between the regulatory processes of NVC and dCA and found that dCA efficiency is reduced during active NVC in healthy older adults (Beishon L. C. et al., 2021). The elucidation of the interrelationships among dCA, DVR, and NVC represents one of the aims of the present study.

Several central mechanisms also impact the regulation of cerebral perfusion with effects that are intertwined with those of NVC, dCA, and DVR. Such central mechanisms include the chemoreflex and baroreflex (Battisti-Charbonney et al., 2011; Marmarelis et al., 2020), as well as hypoxia-driven (Govindan et al., 2019) and metabolic/endocrine mechanisms (Hosford et al., 2022) that operate at a systemic level. The integrated effects of all these regulatory mechanisms, which are coordinated by the autonomic nervous system (Willie et al., 2014), remain a subject of utmost importance to which the present study aspires to contribute.

NVC can be measured indirectly using a range of techniques including magnetic resonance imaging, positron emission tomography, and single photon emission computed tomography (Beishon et al., 2021a). Bedside techniques such as near-infrared spectroscopy (NIRS) and transcranial Doppler ultrasonography (TCD) are increasingly used to measure NVC indirectly, given they are non-invasive and portable (Phillips et al., 2016). TCD uses ultrasound to measure real-time changes in cerebral blood velocity (CBv) in the anterior, middle, or posterior cerebral arteries (ACA, MCA, or PCA, respectively) (Panerai, 2008; Panerai, 2009). In terms of NVC, a range of paradigms have been used to study beat-to-beat changes in CBv upon neuronal activation including sensory (visual, auditory), motor, and cognitive (Stroobant and Vingerhoets, 2000; Beishon et al., 2017; Beishon et al., 2018c; Beishon et al., 2021a). Different paradigms evoke changes in CBv to varying extents depending on the stimulation protocol, study design, and vessel activation (Stroobant and Vingerhoets, 2000).

Previously, we characterized CBv responses induced by cognitive tasks from the Addenbrooke Cognitive Examination III (ACE-III) (Beishon et al., 2018c). The ACE-III is a cognitive assessment tool used to support clinical diagnosis of dementia in a range of settings. The test comprises 20 cognitive tasks covering five cognitive domains: attention, memory, verbal fluency, language, and visuospatial (Hsieh et al., 2013). The present study analyzes data only from attention, fluency and memory tasks. In our previous work, we also characterized the CBv response dynamics using coherent averaging and multivariate time-domain analysis (Barnes et al., 2022) under the assumptions of linearity and stationarity, although these assumptions may be questioned in some cases (Placek et al., 2017; Marmarelis et al., 2014). Frequency-domain approaches have also been used extensively to study dCA through Transfer Function analysis (Claassen et al., 2016; Panerai et al., 2023). Alternative time-domain approaches, such as kernel-based and Principal Dynamic Mode (PDM) analysis (Mitsis et al., 2004; Marmarelis et al., 2013; Marmarelis et al., 2014; Marmarelis et al., 2017; Marmarelis et al., 2019; Marmarelis et al., 2020; Marmarelis et al., 2022) and other time-domain methods (Liu et al., 2015; Phillips et al., 2016; Beishon et al., 2018a; Govindan et al., 2019; Beishon et al., 2021b) may offer some advantages in the study of dCA and/or DVR. However, their use in NVC studies has yet to be explored.

In our previous work (Beishon et al., 2018c), all of these cognitive stimuli resulted in an increase in CBv to varying extents in healthy individuals in the middle cerebral artery. However, not all individuals produce a response to stimulation (Beishon et al., 2020), and the reasons for this are not clear but they may pertain to the “vasodilatory reserve” (see Discussion). In patients with mild cognitive impairment and Alzheimer’s disease, we identified a reduced response to cognitive stimulation, relative to healthy older adults (Beishon et al., 2021b).

In this study, we aimed to analyze changes in the regulation mechanisms of CBv that are stimulated via cognitive paradigms of the attention, fluency, and memory domains from the ACE-III database, relative to the resting state, using kernel-based and PDM approaches, and to compare the results derived using these methods with previously obtained results.

## 2 Materials and methods

### 2.1 Data collection

Forty healthy volunteers were recruited at the University of Leicester, aged over 18 years, free from major disease, and excluding pregnant or breastfeeding women. Demographics on age, sex, handedness, smoker status, height, weight, BMI, alcohol consumption, and ACE-III scores for the subset of the ACE-III cohort analyzed in this study (n = 39) are shown in Table 1. Subjects with stable, well controlled medical conditions (e.g., diabetes, hypertension) were included. Well-controlled comorbidities were stable on and off medication (<140/90 mmHg for controlled hypertension or established on treatment; stable glycaemic control on or off anti-diabetic medication for diabetics). Subjects also confirmed that their medical conditions were controlled and stable. Brief descriptions of the cognitive tasks from the ACE-III protocol that were analyzed in this study are given in Table 2, along with the mean (SD) of the time to complete each task.

**TABLE 1:** Demographics for the subset of the ACE-III cohort (n = 39) analyzed in this study.

	N	Percentage (%)
Sex (F/M)	26/13	66.7/33.3
Handedness (R/L)	36/3	92.3/7.7
Smoker	0	0
Ex-Smoker	4	10.3
Non-Smoker	35	89.7

	Mean	Standard deviation
Age (years)	36.0	14.9
Weight (kg)	70.7	14.9
Height (cm)	169.1	9.4
BMI (kg/m^2^)	24.6	4.8
Units of alcohol per week	7.2	5.5
ACE-III score^a^	97.9	2.1

^a^ Typically, ACE-III scores are compared against a threshold to determine whether a diagnosis of dementia should be considered. Normal cognition is cut off at 88 and above while anything below 82 indicates cognitive impairment. Scores ranging between 83 and 87 are considered inconclusive (Beishon et al., 2019; Bruno and Vignaga, 2019). However, it should be noted that researchers are generally free to shift these ranges depending on the purpose of the research.

**TABLE 2:** Summary of attention, fluency, and memory tasks chosen from the ACE-III protocol alongside mean (SD) of time to complete tasks as a measure of subjects’ performance.

Task #	Domain	Details	Task duration (s)
1	Attention	Orientation to time (day/date/month/year/season)	15.05 (3.27)
2	Attention	Orientation to space (floor/hospital/city/county/country)	14.38 (2.02)
3	Attention	Repeat and remember 3 words (lemon/key/ball)	14.41 (1.69)
4	Attention	Subtract serial sevens from 100	27.24 (11.08)
5	Memory	Recall the 3 words learnt earlier (Task #3: lemon/key/ball)	7.62 (4.42)
6	Fluency	Naming as many words beginning with “P” in 1 min (verbal)	94.51 (4.17)
7	Fluency	Naming as many animals in 1 min (verbal)	74.08 (5.25)
8	Memory	Learn and remember a name and address	61.84 (10.16)
9	Memory	Names of current and previous United Kingdom prime ministers and US presidents	26.08 (6.02)

Note that the latter calculations include only 38 of the 39 subjects for whom task durations were available.

Measurements were made at the Cerebral Haemodynamics in Ageing and Stroke Medicine (CHiASM) research space at the Leicester Royal Infirmary. The CHiASM lab is a quiet, temperature-controlled environment, free from distraction. Continuous measurements of beat-to-beat CBv (TCD, Viyasis Companion III), arterial blood pressure (ABP, Finometer, Finapres Medical Systems, Amsterdam), end-tidal CO_2_ (EtCO_2_, nasal capnography via Capnocheck Plus), and heart rate (HR, 3-lead ECG) were recorded. Subjects underwent a 5-minute resting, baseline measurement while seated, followed by 20 tasks from the ACE-III protocol, with one-minute rest between tasks to allow CBv to return to baseline. In our previous work, we used a 30 s period of rest between tasks. However, not all subjects returned to baseline during this timeframe, particularly older subjects. Therefore, to ensure resting baseline was reached between tasks this was extended to 1 min (Beishon et al., 2018c).

For this study, we analyzed the CBv response to 9 tasks in the attention, fluency, and memory categories (see Table 2). The other task categories (language and visuospatial/visuomotor) activate other areas of the brain besides the prefrontal cortex, the area that we have turned our attention to when measuring cerebral perfusion, and were therefore excluded for our purposes. It has been previously noted that different tasks produce different levels of activation (Beishon et al., 2017; Beishon et al., 2018c), however it has also been reported that the CBv response is not impacted by variations in task duration and task complexity (Intharakham et al., 2022). The selected 9 tasks were recorded contiguously and the analyzed input-output time-series data compose a single continuous data-record, which is a requirement of the employed LET methodology. The beginning of each task was marked using an event recorder. Signals were recorded at 500 Hz and data were stored in the PHYSIDAS data acquisition software for offline analysis.

The vast majority of the participants scored correctly on all or most tasks with a mean score of 98 out of 100 (Table 1). Individual performance data for each task is not included as this study is re-analysis of a previously obtained dataset using a different method of analysis, however no one in the cohort scored below the threshold (88), which qualifies them all as cognitively normal (Beishon et al., 2018c). For the purposes of this study, the successful performance of the task is less important than the presence of cognitive activity.

Data collection methods are described in full in previously published reports (Beishon et al., 2017; Beishon L. et al., 2018; Beishon et al., 2018c). This study was conducted with full approval by the University of Leicester Ethics Committee (Reference: 5355-vjh12-cardiovascularsciences) in accordance with STROBE guidelines and the current version (2008) of the Declaration of Helsinki of 1975. All subjects provided written informed consent.

### 2.2 Data preprocessing

The preprocessing procedure has been described in previous publications (Marmarelis et al., 2017; Marmarelis et al., 2020) and summarized below. The ABP signal was downsampled to 25 Hz after low-pass filtering with a Hanning window (+/−8 samples) to reduce high-frequency random fluctuations. Local minima were detected in order to demarcate the R-R interval of the cardiac cycle that defines each ABP pulse. The average value of each ABP pulse is the “mean ABP” that was placed at the midpoint of the respective R-R interval. To obtain evenly sampled data at 2 Hz, we used cubic spline interpolation. The resulting evenly sampled ABP time-series data were clipped at +/−2.5 standard deviations before processing. The recorded CBv signal was filtered with a Hanning window (+/−15 samples) to smooth the intrapulse waveform. CBv pulses were demarcated with R-R intervals and averaged, resulting in “mean CBv” values for each heart-beat, which were subsequently resampled at 2 Hz using cubic spline interpolation to yield evenly sampled CBv time-series data that are contemporaneous with the ABP data. The CO_2_ signal was downsampled to 10 Hz after smoothing with a Hanning window (+/−5 samples), and the maxima of its first difference were used to mark the beginning of each successive breath. The maximum CO_2_ value for each breath was considered the end-tidal CO_2_ (EtCO_2_) and was placed at the midpoint of the respective breath interval. The final preprocessed EtCO_2_ time-series data resulted from even sampling at 2 Hz, using cubic spline interpolation, and subsequent clipping at +/−2.5 standard deviations. One subject was dropped due to poor data quality, resulting in inability to extract the above described time-series data.

### 2.3 Kernel-based analysis with the Laguerre expansion technique (LET)

**FIGURE 1:**
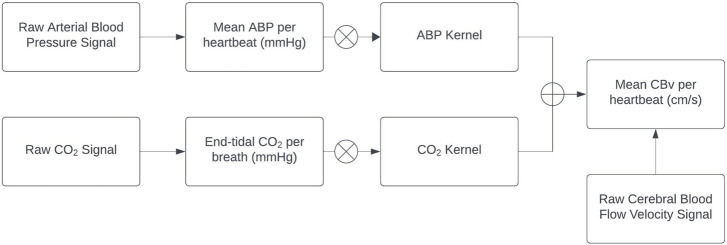
Block diagram of the employed linear time-invariant model with ABP and EtCO_2_ inputs (extracted from the raw signals) convolving the respective kernels to generate the model-predicted CBv output as the sum of the two convolutions (denoted by the Ⓧ symbol). The ABP and EtCO_2_ kernels describe the linear dynamics of each input-output pathway and are estimated via LET.

The preprocessed ABP and EtCO_2_ signals are used as inputs to the general linear time-invariant dynamic model composed of the sum of two convolutional terms (see Appendix), while the preprocessed CBv signal is used as the output (Figure 1). The modeling task pertains to the estimation of the two kernels (one for each input-output pathway) using input-output time-series data. The Laguerre Expansion Technique (LET) was introduced by the Marmarelis lab 30 years ago in order to improve the estimation robustness of such kernels of input-output dynamic models using noisy and relatively short data records (Marmarelis, 1993). The application of LET to physiological system modeling has been presented in many previous publications (e.g., Marmarelis, 2004 and references cited therein), and the basic procedure is summarized in the Appendix. The key idea is that the kernel functions (also known as “Impulse Responses”) that define the input-output convolutional models are expanded on the orthonormal basis of Discrete Laguerre Functions (DLFs), thus reducing the number of free parameters in the model that must be estimated. This compaction of the model representation (in terms of number of free parameters) yields considerable advantages in terms of estimation robustness when noisy input-output data are used, as demonstrated in the aforementioned publications. This method is also extendable to multivariate and nonlinear systems/models. In the linear time-invariant case of the present study, there is one kernel for each input-output pair. Discrete Fourier Transform of each kernel yields an estimate of the Transfer Function of the respective input-output pair. Each output is the sum of the convolutions of its inputs with their respective kernels. LET yields robust estimates of the subject-specific kernels that quantify the dynamic relationship of each input with the output and provide a predictive model for any given set of inputs.

### 2.4 Principal dynamic mode analysis

The concept of Principal Dynamic Modes (PDMs) was introduced in order to facilitate the interpretation of the estimated dynamic models. The PDMs are computed via Singular Value Decomposition (SVD) of a rectangular matrix formed by all baseline kernel estimates of the respective input-output pair of all control subjects in a cohort. Each PDM is defined (in ranked order) by the linear combination of the DLFs using the elements of the respective singular vector as coefficients. This approach allows the comparison of kernels obtained under different conditions in terms of their respective PDM expansion coefficients (termed “PDM gains”), which facilitate kernel comparison using the respective vector of PDM gains and may lend themselves to physiological mechanistic interpretation. This way, we can compare the relative effects of each condition (or disease) upon the physiological mechanism(s) represented by the respective PDMs in each subject.

When the various kernel estimates from a given cohort are viewed as linear combinations of a fundamental set (basis) of orthogonal functions (the PDMs), then the latter represent the fundamental dynamic components of each kernel for the given cohort and offer the fastest convergence in explaining (i.e., predicting) the output in terms of the respective input. That is to say, the 1^st^ PDM predicts most of the output signal in terms of the respective input (via convolution) for the given cohort. The next orthogonal component of the cohort kernels that predicts most of the remaining output signal is the 2^nd^ PDM, and so on and so forth. Thus, the kernel expansions in terms of the PDMs are not an arbitrary mathematical/computational construct but the expansion of fastest convergence as dictated by the input-output data of a given cohort (i.e., the dynamic characteristics of each physiological system). In addition to this “natural decomposition” of the kernels in terms of the PDMs, we posit that the PDMs may correspond to specific physiological mechanisms (solely or in combination) that subserve the respective input-output relationship–thus potentially offering a unique and powerful way to interpret the obtained models in terms of causal mechanisms that are involved in defining each input-output relationship. The eventual acceptance of this novel, but more complicated, approach by the peer community will depend on the actual benefits that may accrue from its application (see Discussion).

### 2.5 dCA and DVR model-based indices

In order to examine the statistical significance of the average changes in dCA and DVR between two conditions, we must reduce the estimated model (i.e., kernels) for each subject to scalar indices (Marmarelis et al., 2017; Marmarelis et al., 2019; Marmarelis et al., 2020). This study seeks to achieve this by use of the kernel expansion coefficients of the PDM basis. However, previous studies have used the following definitions based on the respective step responses:

• for dCA, the difference between the maximum value of the ABP-induced step response minus the value at the steady-state (30 s lag), normalized by the maximum value;
• for DVR, the average value of the CBv model-predicted response to a step change in CO_2_ (1 mmHg) over the first 30 s.

The rationale for this definition of the dCA index is that it represents the extent of CBv dynamic reduction due to the dCA mechanism following an ABP step increase. However, this does not account for the observed differences in the time-constants of the dCA-mediated reduction of the CBv response. To address the latter concern, previous studies have used the Gain and Phase Functions of Transfer Function representation of each input-output pair (Claassen et al., 2016; Panerai et al., 2012) and defined frequency-domain dCA indices as:

• the ratio of the average Gain Function values over the range [0.02 Hz, 0.07 Hz] to the average Gain Function over the range [0.07 Hz, 0.15 Hz];
• the average Phase Function over the range [0.02 Hz, 0.07 Hz].

The rationale for the aforementioned time-domain definition of the DVR index is that it represents the average CBv change over the first 30 s due to a step increase of EtCO_2_ (1 mmHg), which is deemed driven by the DVR mechanism. The present study explores the use of PDM gains as dCA and DVR vector indices that can compare the subject kernels under different conditions and may offer insight into the physiological mechanisms affected by different conditions.

## 3 Results

The mean (SD) of the time-average and variability (root-mean-square, RMS) values of the demeaned time-series data) for ABP, EtCO_2_, CBv, HR, and respiration rate (RR) are reported in Table 3, along with the *p*-values for *paired t*-tests between resting and active states. All mean differences of the time-averages are significant (*p* < 0.05), with the mean increase for ABP and HR suggesting greater sympathetic outflow during intense cognitive activity. All mean differences of the variability of these physiological signals are strongly significant (*p* < 10^–6^).

**TABLE 3:** Mean (SD) of time-averages of the ABP, EtCO_2_, CBv, HR, and RR time-series data, as well as mean (SD) of signal variability quantified by the root-mean-square (RMS) values of the de-meaned ABP, EtCO_2_, CBv, and HR time-series data of the cohort under baseline (BL) and cognitively active (COG) conditions.

	ABP (mmHg)		EtCO_2_ (mmHg)		CBv (cm/s)		HR (bpm)		RR (s^-1^)	
	BL	COG	BL	COG	BL	COG	BL	COG	BL	COG
Mean (SD) of time-average value	91.49 (12.70)	96.11 (12.92)	37.28 (2.76)	36.03 (2.26)	52.13 (10.58)	53.49 (10.02)	71.41 (7.80)	77.19 (8.43)	0.26 (0.06)	0.29 (0.05)
*p*-value	**0.0002**		**<0.0001**		**0.023**		**<0.0001**		**0.0002**	
Mean (SD) of RMS value	5.07 (1.50)	7.59 (1.97)	1.03 (0.44)	2.35 (0.73)	3.38 (0.94)	4.77 (1.17)	4.32 (1.99)	7.36 (2.61)	0.27 (0.06)	0.32 (0.04)
*p*-value	**<0.0001**		**<0.0001**		**<0.0001**		**<0.0001**		**<0.0001**	

Significant *p*-values from paired t-tests are bolded. ABP, arterial blood pressure; CBv, cerebral blood velocity; EtCO_2_, end-tidal CO_2_; HR, heart rate; bpm, beats per minute; RR, respiration rate.

**FIGURE 2:**
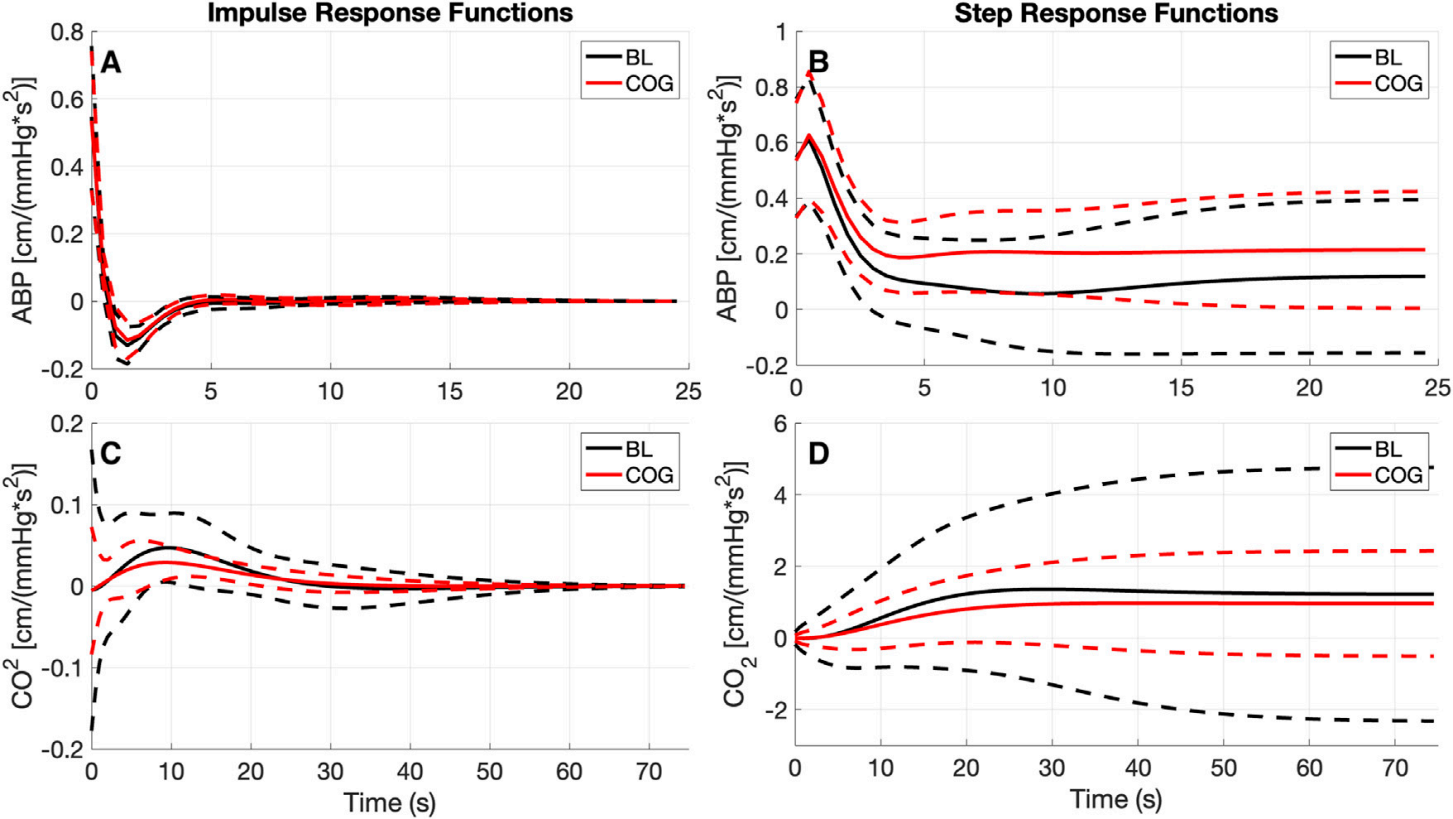
The obtained average kernel estimates **(A, C)** and model-predicted step responses **(B, D)** [in cm/(mmHg*s^2^)] for the ABP-to-CBv **(A, B)** and CO_2_-to-CBv **(C, D)** relations under baseline and active conditions with standard deviation bounds (dashed lines). **(C)** Notable changes are seen only in the size of the CO_2_ kernel that is reduced significantly during cognitively active conditions (COG), while retaining the basic waveform.

Using LET, the obtained average kernel estimates for both inputs under baseline resting conditions (BL) and during cognitive tasks (COG) are shown in Figure 2 (left panels), along with their SD (±σ) bounds. The observed changes in the average kernel estimates for the two input-output dynamic relations between BL and COG conditions indicate subtle changes for the ABP-to-CBv kernel and some amplitude reduction for the CO_2_-to-CBv kernel during COG, while the waveforms remain similar. In order to illustrate the model-predicted effects on CBv of step changes in the two inputs, we show in Figure 2 (right panels) the simulated step responses (i.e., the integrated kernels) for each of the inputs, while the other input is kept at baseline zero. We observe the anticipated effects of dCA in the form of rapid reduction of CBv response after an early peak, relaxing to a low steady-state value (top-right panel) and of DVR in the form of a monotonic rise of CBv response, approaching a high steady-state value (bottom-right panel). The average CBv response to an ABP step change is reduced faster under BL conditions and reaches a lower steady-state value, while the response to a CO_2_ step change reaches a slightly larger steady-state value under BL conditions (see Discussion).

**FIGURE 3:**
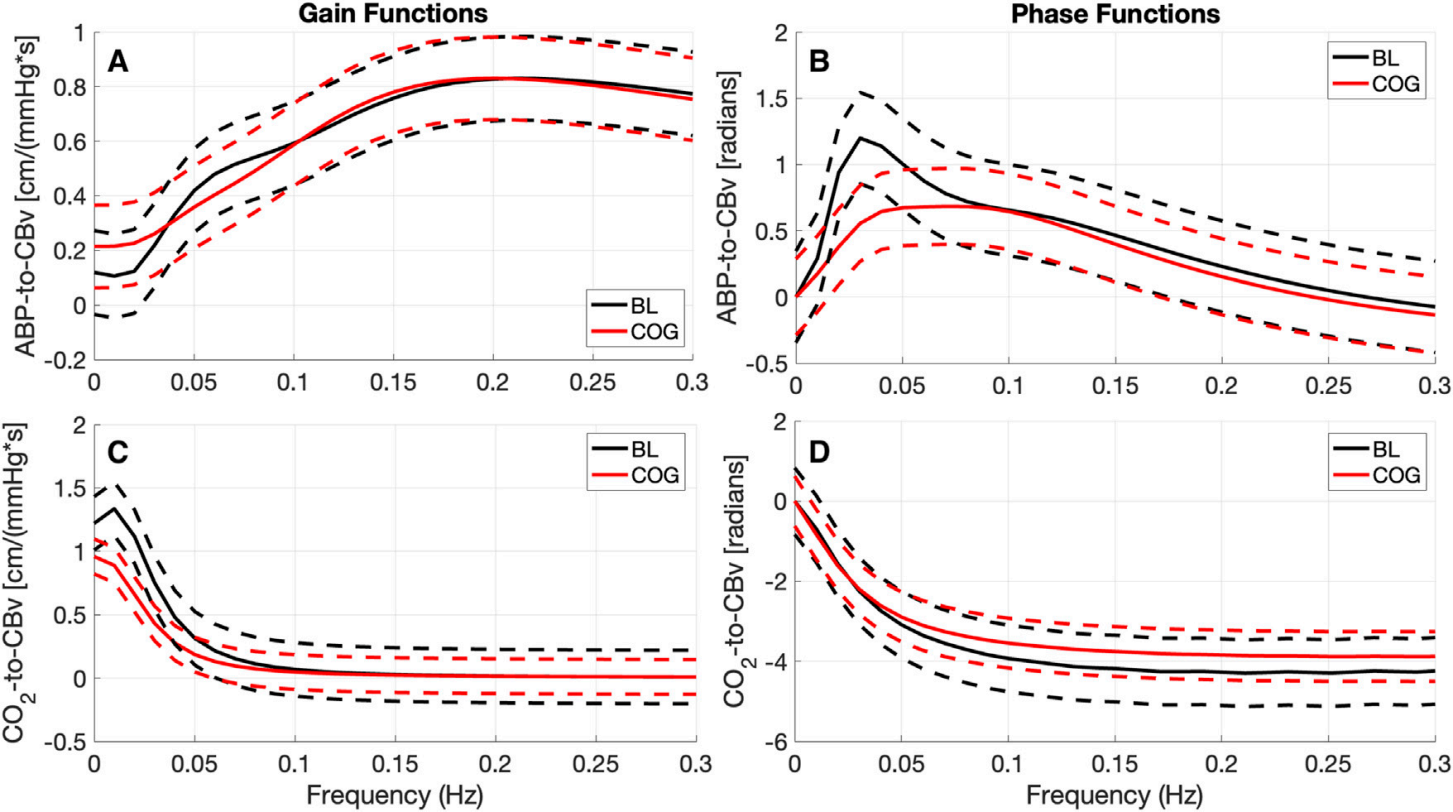
Average Gain Functions **(A, C)** [in cm/(mmHg*s)] and average Phase Functions **(B, D)** [in radians] for the ABP-to-CBv kernels **(A, B)** and CO_2_-to-CBv kernels **(C, D)** under resting (BL) and active (COG) conditions with standard deviation bounds (dashed lines). **(A)** The average ABP Gain Function exhibits lower values below 0.04 Hz and a small bulge around 0.07 Hz for BL conditions. Both average Gain Functions exhibit a primary resonant peak around 0.2 Hz and have similar magnitude. **(C)** The average CO_2_ Gain Functions exhibit similar low-pass characteristics, but slightly reduced under COG condition. The maximum value of the ABP Phase Function **(B)** is reduced considerably for the COG condition.

The changes in the ABP-to-CBv dynamic relation between BL and COG are more clearly discerned in the average Gain and Phase Functions (see Figure 3 top panels). The average Gain Function exhibits a small bulge around 0.07 Hz for BL conditions (not present in COG), while higher values are seen for frequencies below 0.04 Hz for COG. There is a resonant peak in the range 0.20–0.2 Hz (and of similar magnitude) for both conditions. The average CO_2_-to-CBv Gain Function exhibits similar low-pass characteristics for both conditions, but the magnitude is reduced slightly under COG conditions (as in the kernels). There is a notable reduction of the average Phase Function over the range 0.02–0.07 Hz in the COG condition (see top-right panel of Figure 3) that indicates altered vascular compliance over these frequencies where much of the ABP power resides.

Use of the previously defined time-domain indices for dCA and DVR indices, and the frequency-domain indices for dCA yield the results reported in Table 4. Only the time-domain dCA and Phase-Average indices yield significant mean differences (*p* < 0.05), with the Phase Average being a stronger differentiator (i.e., having much smaller *p*-value). The average DVR index is reduced in the COG condition, but this reduction does not reach statistical significance.

**TABLE 4:** Mean (SD) of time-domain and frequency-domain indices for dCA and DVR under resting and active conditions.

Variable	Resting mean (SD)	Active mean (SD)	*p*
dCA (unitless)	0.85 (0.35)	0.67 (0.24)	**0.014**
DVR (in cm/s)	0.71 (0.62)	0.47 (0.37)	0.08
dCA Gain Ratio (unitless)	1.80 (0.54)	2.08 (0.64)	0.11
dCA Phase Average (in radians)	−0.47 (0.59)	−0.88 (0.25)	**<0.0001**

Significant *p*-values are bolded.

**FIGURE 4:**
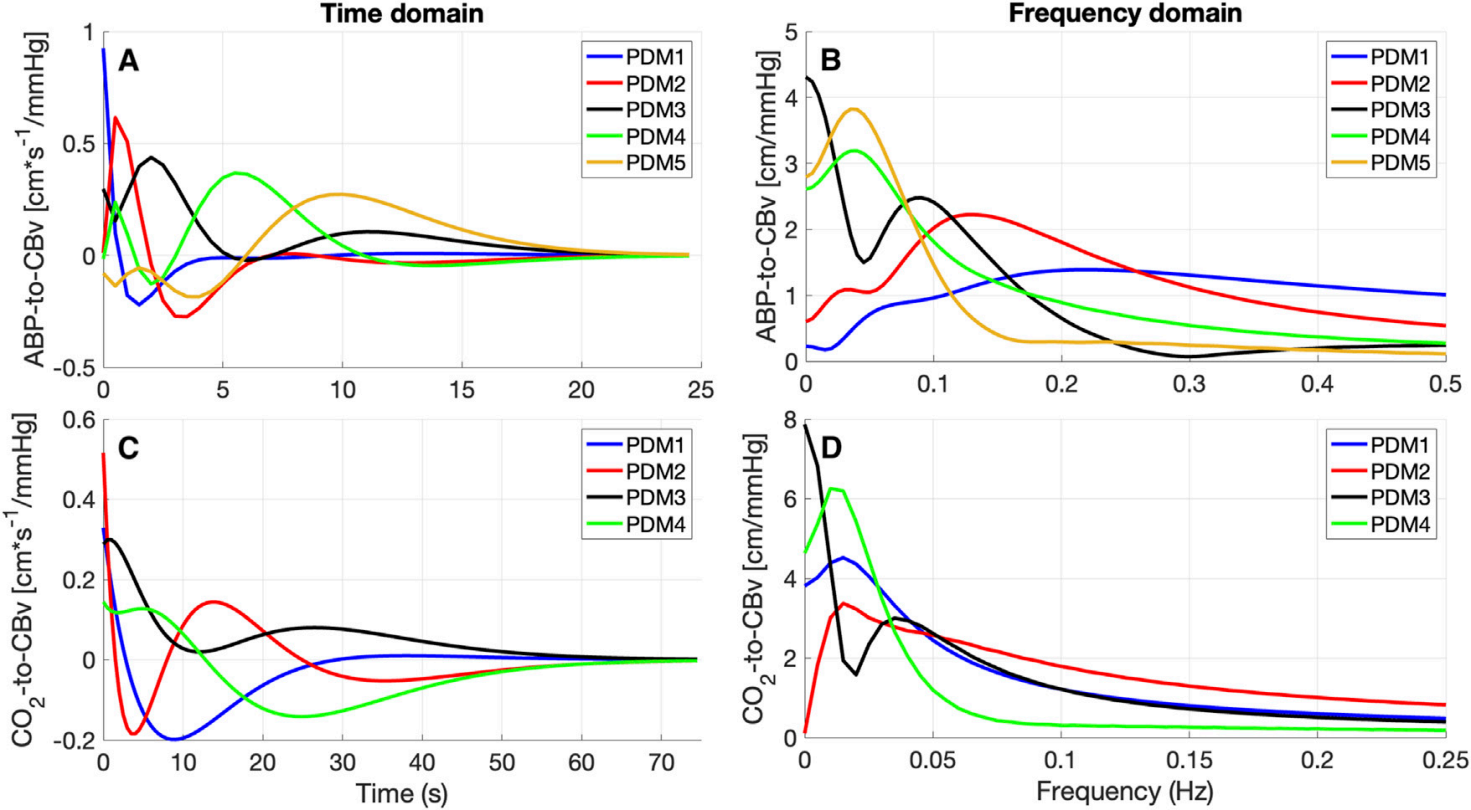
PDMs for the ABP-to-CBv relation **(A, B)** and CO_2_-to-CBv relation **(C, D)** in the time-domain **(A, C)** and their frequency-domain counterparts, magnitude only **(B, D)** [in cm*sec^-1^/mmHg].

The obtained PDMs are shown for the ABP-to-CBv and CO_2_-to-CBv relations in Figure 4, in the time and frequency domains. We see that most PDMs exhibit characteristic resonant peaks (see Discussion). The mean (SD) values of the PDM expansion coefficients (termed “PDM gains”) are shown in Table 5 for both conditions, along with their respective singular values and *p*-values. Only the gains of the 4^th^ and 5^th^ PDM gains for the ABP input were found to be significantly different (*p* < 0.05) for the two conditions (BL vs. COG), suggesting their possible association with NVC activation (see Discussion). The difference for the 3^rd^ PDM gain of the ABP input was borderline significant (*p* = 0.050), inviting some thoughts about its mechanism of origin (see Discussion).

**TABLE 5:** Mean (SD) of PDM gains for ABP and EtCO_2_ inputs, as well as corresponding singular values and *p*-values of comparison of BL vs COG conditions. Significant *p*-values are bolded.

	ABP				EtCO_2_			
Gain #	Singular values	Resting mean (SD)	Active mean (SD)	*p*	Singular values	Resting mean (SD)	Active mean (SD)	*p*
1	3.95	0.59 (0.22)	0.57 (0.22)	0.454	1.87	−0.19 (0.23)	−0.12 (0.11)	0.075
2	0.53	0.01 (0.08)	0.04 (0.07)	0.110	1.49	0.06 (0.23)	0.03 (0.09)	0.406
3	0.31	−0.006 (0.05)	0.01 (0.03)	0.050	1.33	0.08 (0.20)	0.06 (0.08)	0.699
4	0.26	0.005 (0.04)	0.04 (0.03)	**0.0001**	0.88	0.03 (0.14)	0.003 (0.07)	0.362
5	0.17	0.001 (0.03)	−0.02 (0.04)	**0.016**	N/A	N/A	N/A	N/A

## 4 Discussion

The regulation of cerebral blood flow is achieved through intricate coordination of multiple homeostatic mechanisms driven by mechanical signaling (e.g., blood pressure variations) and/or chemical signaling (e.g., blood gas variations) occurring systemically throughout the cerebrovascular and autonomic nexus (Willie et al., 2014). These two types of regulatory processes correspond to the widely recognized mechanisms of dCA and DVR. In addition, neurovascular coupling (NVC) is driven by chemical signals released from perivascular nerves and astrocytes in order to modulate cerebrovascular tone and adjust cerebral perfusion to the localized needs of brain activity (Hamel, 2006; Atwell et al., 2010; Phillips et al., 2016). The actions of NVC, dCA, and DVR are intertwined in seeking homeostatic regulation of cerebral perfusion and will have to be studied in a dynamic context in order to achieve a comprehensive understanding of this dynamic physiological function. The study of the dynamics of this nexus of regulatory mechanisms is very complex, but also extremely important in advancing our understanding of the regulation of cerebral perfusion that impacts multiple critical aspects of brain function in health and disease. The present study seeks to introduce an advanced data-based method for the robust quantitative modeling of some key aspects of the dynamics of cerebral perfusion (i.e., dCA, DVR, and NVC) in order to address the complexity of these dynamic mechanisms. Previous studies have largely examined these mechanisms in isolation, rather than in interaction with each other, with some notable exceptions that examined interactions between two of them (Beishon L. C. et al., 2021; Marmarelis et al., 2017), but not all three.

Some previous quantitative studies of the effects of cognitive activity on cerebrovascular function and key hemodynamic variables have utilized time-averages of the physiological measurements that capture static (not dynamic) relationships among these variables (e.g., Lefferts et al., 2018; Beishon et al., 2018a; Beishon L. et al., 2018). The latter studies analyzed changes in the time-averages of continuously recorded physiological signals that are generally consistent with our findings reported in Table 3 – i.e., the reported time-average values are affected significantly by cognitive activity for all recorded physiological signals, based on *paired* t-tests comparing the changes for each subject under the two conditions (BL vs. COG). Note that, at the group level (*unpaired* t-tests), the *p*-values are larger. The significant increases of DC values for systemic ABP, HR, and RR are expected due to increased sympathetic outflow associated with the performance of cognitive tasks, independently of NVC activation. The significant EtCO_2_ decrease is probably associated with the hyperventilation resulting from the increased RR during cognitive exertion. Table 3 also shows significant increases of variability (RMS value of demeaned signals) for all variables in the COG state, which may be attributed to autonomic and/or NVC activation associated with the cognitive tasks. It must be emphasized that static analysis pursued through regression modeling seeks to discover possible *correlations* between the endogenous (dependent) and the exogenous (independent) variables (e.g., Lefferts et al., 2018). The study of the dynamics of this system was initially performed through direct observation of the time-course of changes in CBv in response to cognitive, ABP, and CO_2_ stimulation, usually in pulsatile form and occasionally including motor stimulation (Panerai et al., 2000; Panerai et al., 2005; Panerai et al., 2012; Salinet et al., 2013; Phillips et al., 2016; Beishon et al., 2017; Beishon et al., 2018a). Dynamic modeling seeks to reveal and quantify the precise effects of input changes upon output changes in a continuum of time to allow prediction; thus, it is more ambitious in terms of the amount of new knowledge that it seeks to extract (i.e., temporal patterns of dynamic effects) and, inevitably, requires more sophisticated/complicated methods of analysis, such as the kernel estimation method via LET that is used in the present study. A similar approach, using kernel expansions, was reported in the study of regional NVC response to changes in arterial CO_2_ through functional MRI (fMRI) BOLD data (Prokopiou et al., 2019) and showed that different brain regions exhibit distinct patterns of such dynamics. A second layer of generated knowledge results from the PDM analysis presented herein that pertains to dominant *dynamic components* within the kernels of a given cohort–naturally requiring additional sophisticated methods of analysis. The rationale for these more demanding methods of dynamic analysis is that they yield new and valuable insights into the physiological systems of interest, which cannot be reached with static methods.

Previous studies of NVC dynamics include a specialized software package to investigate the time-course of changes in BOLD measurements of fMRI during specific visual stimulation protocols (Phillips et al., 2016). These can elucidate our understanding of NVC dynamics but more often than not they fail to provide a generalizable dynamic modeling approach. Other studies opted to use Auto-Regressive Moving-Average (ARMA) modeling to examine the dynamics of cerebral perfusion during a motor task in a multivariate context including the effects of hypercapnia (Panerai et al., 2012; Maggio et al., 2014; Barnes et al., 2022). Notable is also the use of Transfer Function analysis in the study of regional DVR through fMRI BOLD data (Duffin et al., 2015). The dynamic modeling used in the present study seeks to reveal the precise convolutional pattern (kernels) by which changes in either of the two input variables cause changes in the output variable (see Equation (A1) of Appendix). This objective is shared by ARMA modeling and Transfer Function analysis, but these three approaches differ in terms of estimation robustness in the presence of high noise in the input-output data.

The results of the kernel-based analysis of this study (see Figure 2), indicate a slight reduction in the size of the average CO_2_-to-CBv kernel during COG condition (without noticeable change in waveform) that can be attributed to competition of the activated NVC towards the mechanism of CO_2_ vasoreactivity, as they both seek vasodilation within the constraints imposed by the maximum natural extendibility of the vessels (Panerai et al., 2000; Panerai et al. 2005; Panerai et al. 2012; Maggio et al., 2014; Hosford et al., 2022). This has been regarded as a reduction in “*vasodilatory reserve*” (Willie et al., 2014; Prokopiou et al., 2019). Nonetheless, no statistical significance was found in this study regarding the difference of the DVR indices (marginal *p* = 0.08) or regarding the PDM gains of the CO_2_-to-CBv relation (see Table 5, marginal *p* = 0.07 for 1^st^ PDM) between the BL and COG conditions. This is consistent with the results reported previously on the dynamic effects of cognitive/motor tasks and hypercapnia on DVR (Panerai et al., 2012; Maggio et al., 2014).

The observed changes in the average ABP-to-CBv kernel during COG conditions are subtle and become visually more apparent in the respective Gain Functions (Figure 3, left panel), where the main differences are in frequencies below 0.04 Hz and around 0.07 Hz. The average step response (top-right panel of Figure 2) demonstrates the reduction of dCA during COG, as has been reported previously (Panerai et al., 2012; Maggio et al., 2014; Barnes et al., 2022). It is worth noting that the 3^rd^ PDM gain of the ABP-to-CBv relation shows borderline significant change for COG vs. BL (*p* = 0.050) and increase in magnitude under COG. Importantly, this PDM exhibits a resonant peak close to the resonant frequency of the Mayer wave of vascular tone (around 0.1 Hz) that is associated with sympathetic outflow (Julien, 2006). Therefore, it is plausible that this PDM gain represents the quantitative effects of changed sympathetic activity upon dCA during COG–since sympathetic activation is expected to increase during the exertion of intense cognitive tasks.

Significant differences are revealed in the ABP-to-CBv dynamic relation through PDM analysis, specifically in the 4^th^ and 5^th^ ABP PDM gains (*p* < 0.05, see Table 5) – suggesting possible association of NVC with these two ABP PDMs. These PDMs exhibit a resonant peak around 0.04 Hz, which would be consistent with reported biphasic Hemodynamic Response Functions (HRFs) in functional MRI studies of BOLD signals (Boynton et al., 1996; West et al., 2019) that depend critically on the NVC mechanism. We note, however, that various other HRFs have also been reported for different brain regions and under different stimulation conditions. We also note that the increase in the absolute value of these PDM gains under COG condition suggest enhanced activation of the respective NVC mechanisms. The top-left panel of Figure 2 indicates that the 4^th^ and 5^th^ ABP PDMs are similar in waveform but shifted relative to each other by about 5 s. This can give rise to the hypothesis that they may correspond to the distinct glutamate-driven NVC pathways from neurons or astrocytes, respectively, where the neuron-pathway is preceding in NVC action (Attwell et al., 2010).

In terms of interactions between the dCA, DVR, and NVC mechanisms, our results indicate that COG-driven NVC activity reduces the dCA and DVR (see right panels of Figure 2), although the DVR reduction does not rise to statistical significance. These results are consistent with what was reported previously for cognitive-motor tasks (Panerai et al., 2012). The interactions among these three mechanisms may be viewed in the context of “vasodilatory reserve” and mechanical vascular constraints (Willie et al., 2014; Prokopiou et al., 2019). In general, different tasks may result in different levels of activation, and this has been reported in detail in previous publications (Beishon et al., 2017; Beishon et al., 2018a), although another study did not find any significant effect on CBv due to cognitive task duration or task complexity (Intharakham et al., 2022). The goal of this study was to apply a different method to analyze and interpret the neurovascular coupling response under stimulation from various cognitive tasks that may impact cerebral circulation at the prefrontal cortex.

The presented dynamic modeling methodology may be used for assessing the possible effects of neurovascular and/or neurocognitive pathologies upon changes of cerebral perfusion between cognitively active and resting state in order to assist with clinical diagnosis and discovery of treatment targets for the affected physiological mechanisms. In this regard, the presented PDM analysis holds the promise of quantifying the effects on relevant physiological mechanisms.

In conclusion, this dynamic modeling study indicates significant changes in dCA, but not DVR, during cognitively active state (relative to the resting state) in healthy adults, consistent with previously reported observations on the effects of cognitive-motor task on dCA and DVR. The specific dynamics of these changes, which are mainly due to the increased activation of NVC, are captured by the novel PDM analysis that decomposes the transfer relations underlying dCA and DVR into their main dynamic components in order to identify the physiological mechanisms relevant to dCA and DVR. The observed significant changes in specific PDMs suggest their association with NVC dynamics, consistent with some published HRFs of BOLD data analysis in functional MRI.

## 5 Study limitations

The ε4 allele of the apolipoprotein E gene (APOE4) is recognized as a significant risk factor for developing Alzheimer’s Disease due to its association with astrocytic/microglia activation and alterations of vascular mural cells leading to endothelial disruption and decreased amyloid clearance (van Dijk et al., 2007; Viticchi et al., 2014; Yamazaki et al., 2021). Thus, the presence of APOE4 in some subjects is likely to affect the presented results of our analysis of cerebral perfusion regulation. Unfortunately, APOE genotyping is not available for this cohort and, therefore, our analysis could not take this genetic factor into account.

Additionally, the number of subjects with diabetes and/or hypertension is not known. Knowing that any such conditions are controlled may indeed be useful for our analyses, since the effect of these potential confounders may influence our results.

Finally, the use of TCD imposes certain limitations as 10%–20% of the population (especially older) do not have a proper temporal window to allow this measurement. TCD is also a localized measurement (at the middle cerebral arteries) and constrained in extending the results to cerebral blood flow by the assumption of unchanged cross sectional area of the artery. Finally, the size of the cohort was limited and some of the demographics were skewed (e.g., younger and more educated subjects than the general population). No statistical power calculation was carried out. A larger cohort with more representative demographics in future studies will benefit the statistical robustness of the obtained results.

## Data Availability

The original contributions presented in the study are included in the article/supplementary material, further inquiries can be directed to the corresponding author.

## APPENDIX:The Laguerre expansion technique (LET)

The general formulation of the linear time-invariant dynamic model for a two-input/one-output system is given by Equation (A1) that expresses the output *y(n)* as the sum of two convolutional terms (corresponding to the two inputs *p(n)* and *z(n)* and their respective kernels) plus a constant $k_{0}$ :

$$y\left(n\right)=k_{0}+\sum_{m=0}^{M_{P}}kp\left(n-m\right)\;p\left(m\right)+\sum_{m=0}^{M_{z}}kz\left(n-m\right)\;z\left(m\right)$$
(A1)

where *p(n)* denotes the ABP input, *z(n)* denotes the CO_2_ input, *k*_*p*_ denotes the ABP-to-CFV kernel and *k*_*z*_ denotes the CO_2_-to-CFV kernel. The modeling task seeks the estimation of the two kernels (over all values of their memory extent *M*_*p*_ and *M*_*z*_) from input-output data (*n = 1, … ,N*). To this purpose, we use the Laguerre expansion technique (LET) which has been shown to yield reliable kernel estimates for linear dynamic models with multiple inputs even for noisy and relatively short input-output datasets (Marmarelis, 1993; Marmarelis 1004). According to this approach, each kernel is expanded on a properly selected discrete-time Laguerre function (DLF) basis as:

$$k_{p}\left(m\right)=\sum_{j=1}^{Q_{p}}C_{j}^{p}L_{j}^{p}\left(m\right),k_{z}\left(m\right)=\sum_{j=1}^{Q_{z}}C_{j}^{z}L_{j}^{z}\left(m\right)$$
(A2)

where {*L* ^*i*^_*j*_ *(m)*} (*j = 1, … ,Q*_*i*_) denotes the orthonormal *Q*_*i*_-dimensional DLF basis for the *i*-th kernel (*i* is either *p* or *z* in this study). These kernel expansions transform the input-output relation of Equation (A1) into Equation (A3) that involves *linearly* the unknown Laguerre expansion coefficients { $C_{j}^{p}$ } and { $C_{j}^{z}$ }, which must be estimated for each input-output pair:

$$y\left(n\right)=k_{0}+\sum_{j=1}^{Q_{p}}C_{j}^{p}v_{j}\left(n\right)+\sum_{j=1}^{Q_{z}}C_{j}^{z}u_{j}\left(n\right)$$
(A3)

where:

$$v_{j}\left(n\right)=\sum_{m=0}^{n}L_{j}^{p}\left(n-m\right)\;p\left(m\right),u_{j}\left(n\right)=\sum_{m=0}^{n}L_{j}^{z}\left(n-m\right)\;z\left(m\right)$$
(A4)

are the convolutions of each input with the respective DLF basis. Since the Laguerre expansion coefficients enter linearly in the input-output model of Equation (A3), their estimation can be achieved via least-squares regression (a simple and robust numerical procedure). In this study, LET utilized 5 DLFs for the kernel of the ABP input with Laguerre parameter alpha = 0.5 and 4 DLFs for the kernel of the CO_2_ input with Laguerre parameter alpha = 0.85. The selection of the number of DLFs and alpha values was based on a search procedure that minimized the Bayesian Information Criterion of the average model prediction over the grid of values: *L* = 1 to 8 and alpha = 0.1 to 0.9 (every 0.05).
